# Short- and long-term outcomes of HIV-infected patients admitted to the intensive care unit: impact of antiretroviral therapy and immunovirological status

**DOI:** 10.1186/2110-5820-2-25

**Published:** 2012-07-04

**Authors:** David Morquin, Vincent Le Moing, Thibaut Mura, Alain Makinson, Kada Klouche, Olivier Jonquet, Jacques Reynes, Philippe Corne

**Affiliations:** 1 Medical Intensive Care Unit, Gui de Chauliac Teaching Hospital, University of Montpellier 1, Montpellier, France; 2 Infectious and Tropical Diseases Department, UMI 233, Gui de Chauliac Teaching Hospital, Montpellier, France; 3 Department of Medical Information, Lapeyronie Teaching Hospital, Montpellier, France; 4 Inserm, Clinical Investigation Center 1001, Saint-Eloi Teaching Hospital, Montpellier, France; 5 Medical Intensive Care Unit, Lapeyronie Teaching Hospital, University of Montpellier 1, Montpellier, France; 6 Medical Intensive Care Unit, Gui de Chauliac Teaching Hospital, 80 avenue Augustin Fliche, Montpellier, Cedex 5, 34295, France

**Keywords:** Intensive care units, Human immunodeficiency virus, Acquired immunodeficiency syndrome, Antiretroviral therapy, Prognostic factors, Critical care, Mortality

## Abstract

**Background:**

The purpose of this study was to assess the short- and long-term outcomes of HIV-infected patients admitted to intensive care units (ICU) according to immunovirological status at admission and highly active antiretroviral therapy (HAART) use in ICU.

**Methods:**

Retrospective study of 98 HIV-infected patients hospitalized between 1997 and 2008 in two medical ICU in Montpellier, France. The primary outcome was mortality in ICU. The secondary end point was probability of survival in the year following ICU admission.

**Results:**

Eighty-two (83.6%) admissions in ICU were related to HIV infection and 45% of patients had received HAART before admission. Sixty-two patients (63.3%) were discharged from ICU, and 34 (34.7%) were alive at 1 year. Plasma HIV RNA viral load (VL) and CD4+ cell count separately were not associated with outcome. Independent predictors of ICU mortality were the use of vasopressive agents (odds ratio (OR), 3.779; 95% confidence interval (CI), 1.11–12.861; *p* = 0.0334) and SAPS II score (OR, 1.04; 95% CI, 1.003-1.077; *p* = 0.0319), whereas introducing or continuing HAART in ICU was protective (OR, 0.278; 95% CI, 0.082-0.939; *p* = 0.0393). Factors independently associated with 1-year mortality were immunovirological status with high VL (>3 log_10_/ml) and low CD4 (<200/mm^3^; hazard ratio (HR), 5.19; 95% CI, 1.328-20.279; *p* = 0.0179) or low VL (<3 log_10_/ml) and low CD4 (HR, 4.714; 95% CI, 1.178-18.867; *p* = 0.0284) vs. high CD4 and low VL, coinfection with C hepatitis virus (HR, 3.268; 95% CI, 1.29-8.278; *p* = 0.0125), the use of vasopressive agents (HR, 3.68; 95% CI, 1.394-9.716; *p* = 0.0085), and SAPS II score (HR, 1.09; 95% CI, 1.057-1.124; *p* <0.0001). Introducing HAART in a patient with no HAART at admission was associated with a better long-term outcome (HR, 0.166; 95% CI, 0.043-0.642; *p* = 0.0093).

**Conclusions:**

In a population of HIV-infected patients admitted to ICU, short- and long-term outcomes are related to acute illness severity and immunovirological status at admission. Complementary studies are necessary to identify HIV-infected patients who benefit from HAART use in ICU according to immunovirological status and the reasons of ICU admission.

## Background

Since the highly active antiretroviral therapy (HAART) era, mortality of HIV-infected patients in intensive care units (ICU) has decreased from nearly 90% to 20% [1-9], and reasons for admission of HIV-infected patients in ICU have evolved from AIDS to non-AIDS classifying complications [9,10].

Retrospective cohort studies have shown that prognostic factors of mortality in HIV-infected patients admitted to ICU were acute illness severity, poor functional status, low albumin rate, and respiratory failure requiring mechanical ventilation [2,8,11-14]. No specific HIV characteristics (CD4 cell count value, plasma HIV RNA load, HIV-related diagnosis, or management of antiretroviral therapy) were clearly identified as predictors of ICU mortality [6,8-13,15-21], except in one study where the majority of ICU patients had an AIDS-defining illnesses, in which very low CD4 T-cell count (<50 cells/mm^3^) was found to be associated with ICU mortality [17]. We believe that to consider CD4 value and HIV viral load values separately in an HIV-infected population admitted to ICU is not sufficient to assess the prognosis properly.

In the non-ICU HIV-infected population, long-term survival is closely related to both sustained HIV-undetectable viral load under HAART and immune reconstitution [22-24]. In the HIV-infected patients in ICU, four retrospective studies analyzed the impact of HAART and showed discordant results [8,9,17,21]. Moreover, management of HAART in ICU often is challenging because of potential toxicities and drug-to-drug interactions.

The objective of this study was to analyze the impact of immunovirological status and HAART use on short- and long-term outcomes in HIV-infected patients admitted to ICU.

## Methods

### Patients

This study was conducted retrospectively in two medical ICUs, in a 2,300-bed University Hospital in France. All HIV-infected patients admitted between January 1, 1997 and December 31, 2008 were included. Due to the geographic proximity of the Infectious Diseases Department and the constant collaboration of the clinicians, policies of admission of HIV-infected patient in ICU did not change during this period. Clinical admission criteria of HIV-infected patients to ICU did not take into account HIV status.

When a patient was admitted more than once, only the first admission was analyzed. HIV status was either known before admission or discovered during ICU care. Eligible patients were screened by an independent query of the entire ICU database by the investigators and by a cross-computerized search from the Medical Information Department for admission in ICU and any follow-up in the Infectious Diseases Department or the Retrovirology Laboratory Department. All ICU and Infectious Diseases Department medical charts were reviewed by an infectious diseases specialist (DM).

The study design was a retrospective review of patient records in accordance with French law and did not require institutional ethics committee approval. Due to the retrospective design of the study, we did not obtain informed consent from the included patients. Nevertheless, all data collected retrospectively were anonymized in a standardized case-report form and in the database.

### Definitions

AIDS-defining illnesses before ICU admission were classified according to the latest statement of the Centers for Disease Control and Prevention (CDC). HAART was defined as a combination of three or more antiretroviral drugs belonging to at least two classes among the following: protease inhibitors (PIs), nucleoside reverse transcriptase inhibitors (NRTIs), and nonnucleoside reverse transcriptase inhibitors (NNRTIs). Patients were considered as receiving HAART if it was introduced in the previous 30 days or more to admission.

### Data collection and end points

The data were entered into an anonymous customized database (Access 2003; Microsoft) and were subjected to electronic validation rules. Demographic characteristics, comorbidities, biological data (albumin, lactic acid, thrombin time in the first 24 h) and details of life-sustaining treatments used in the ICU (mechanical ventilation, use of vasoactive drugs, renal replacement therapy and durations) were recorded. The SAPS II (Simplified Acute Physiology Score) and APACHE II (Acute Physiology and Chronic Health Evaluation) scores were calculated for each patient within the first 24 h of the ICU stay.

The primary outcome was mortality in ICU. The secondary end point was probability of survival in the year following ICU admission. We looked for prognosis factors of short- and long-term mortality regarding population, ICU disease, and HIV-related characteristics: HAART use before ICU admission, plasma HIV RNA, CD4 count, and HAART introduction or pursuance in ICU.

Plasma HIV RNA and CD4 lymphocyte count measured during the 3 months before or the first week of admission in ICU were used to define four groups:

Patients with no or mild immune deficiency (CD4 lymphocyte cell count ≥200/mm^3^). Subgroups were defined on their plasma HIV RNA viral load (VL) with a threshold at 3 log_10_/ml: [CD4^*high*^ + VL^*high*^] and [CD4^*high*^ + VL^*low*^].

Patients with severe immune deficiency (CD4 lymphocyte cell count <200/mm^3^) Subgroups were defined in the same manner: [CD4^*low*^ + VL^*high*^] and [CD4^*low*^ + VL^*low*^].

The CD4 level threshold of 200/mm^3^ was chosen, because it is predictive of a high risk for opportunistic infections and corresponds to the threshold at which an anti-Pneumocystis and toxoplasmosis prophylaxis is prescribed according to different national recommendations [25]. The plasma HIV RNA VL threshold of 3 logs was arbitrarily chosen to distinguish virological failure from punctual replication under HAART (i.e., “Virologic blip”: after virologic suppression, an isolated detectable HIV RNA at low level that is followed by a return to virologic suppression [26]).

### Statistical analysis

Categorical variables are presented as frequencies with percentages, and continuous variables are presented as means and standard deviation (SD). Patients’ characteristics were compared between the groups by using the chi-square test, Fisher’s exact test, analysis of variance, or Kruskal-Wallis test, when appropriate. Logistic regression analysis was performed to identify predictive factors for ICU mortality and Cox proportional hazard models to identify predictive factors for 1-year mortality after admission in ICU. In both methods, we first calculated crude odds ratio (OR) and hazard ratio (HR) with their 95% confidence intervals (CI) and then adjusted them with multivariate models using a backward selection process with *p* <0.1 used as the cutoff for retention in the models. To avoid collinearity, only one severity scoring system was entered in multivariate models; SAPSII were chosen because the models better fits the data. The times to death during the first year after admission in ICU were compared between immunovirological statuses using the log-rank test and illustrated by Kaplan-Meier survival curves. Statistical analyses were performed at the conventional two-tailed α level of 0.05 using SAS version 9.1 (SAS Institute, Cary, NC). *P* <0.05 was considered significant.

## Results and discussion

### Results

#### Demographic and clinical characteristics of patients

**Figure 1:**
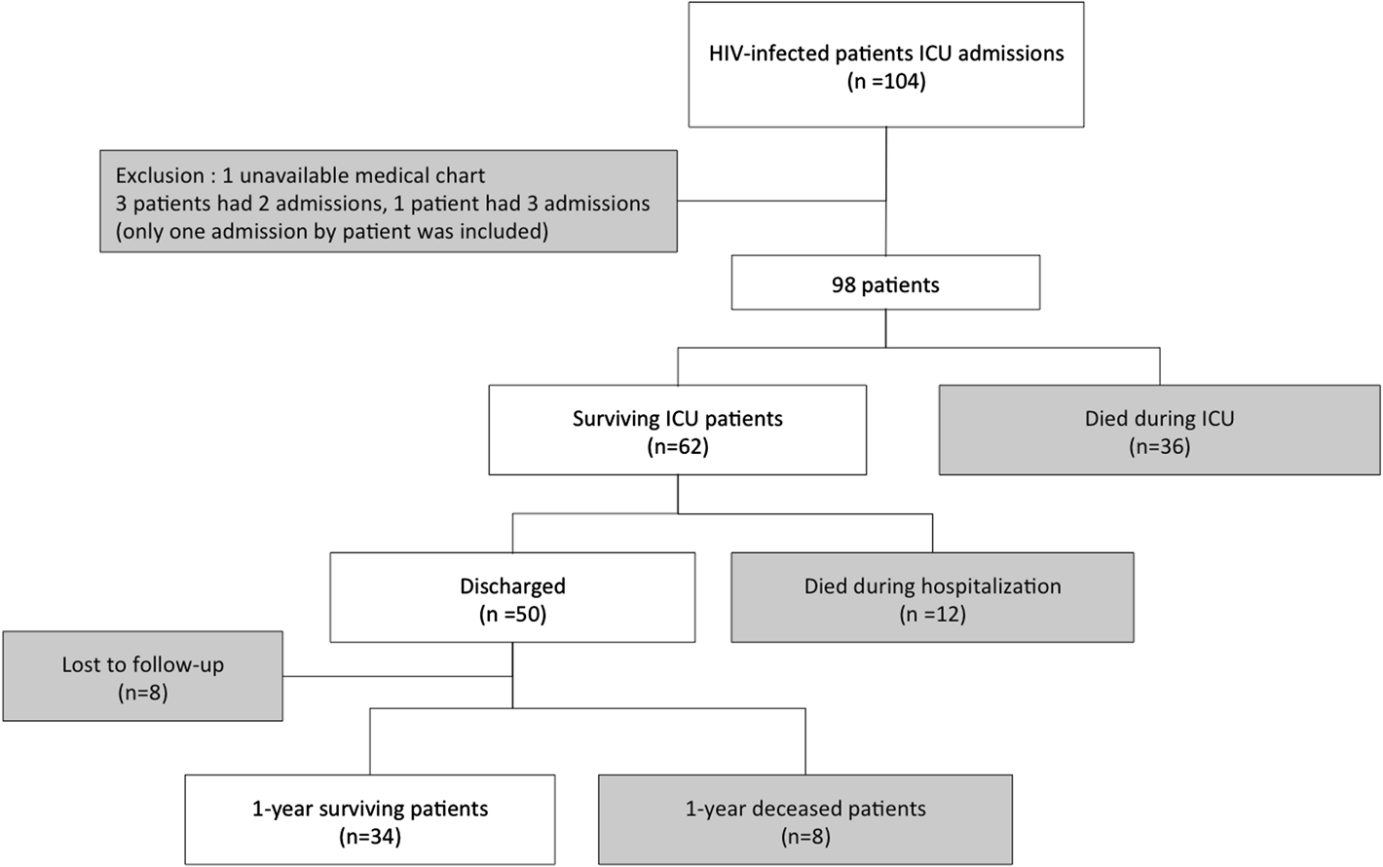
Flow diagram of human immunodeficiency virus (HIV)-infected patients enrolled in the study at Medical Intensive Units of the Montpellier Hospital from 1997 to 2008.

Ninety-nine patients, corresponding to 104 ICU admissions, were screened. One medical chart was missing, and the patient was excluded from analysis. Three patients had two ICU admissions, and one patient had three admissions during the 11-year study period, resulting in 98 admissions of 98 HIV-infected patients analyzed (Figure 1).

The majority of the ICU cohort consisted of men (70.4%). The mean age was 43.1 years, and HIV diagnosis was made more than 5 years before ICU admission for 67 (68.4%) patients and less than 6 months for 17 patients (17.3%). Demographic and clinical characteristics are presented in Table 1. Respiratory and neurological failures were the most common reasons for ICU admission: 39% and 26% respectively. Sixty-three patients (64.3%) required mechanical ventilation, 51 (52%) vasoactive drugs, and 15 (15.3%) renal replacement therapy. Patients had a mean SAPS II score at 53.8 and a mean APACHE II score of 17.5, a mean serum albumin of 31.1 g/L, and a mean serum lactic acid level of 2.9 mmol/l. The mean length of ICU stay was 14 days.

**Table 1:** General characteristics of the 98 HIV-infected patients admitted to the ICU

**Variable (n,%)**		**Total**	**Survived the ICU**	**Died in the ICU**	***p *value**
		**(n = 98)**	**(n = 62)**	**(n = 36)**	
Age, yr (mean ± SD)		43.1 ± 10.7	43.2 ± 10.6	42.9 ± 10.9	0.815
Males		69 (70.4)	44 (71)	25 (69.4)	0.873
**Comorbidities**					
Diabetes		10 (10.2)	8 (12.9)	2 (5.6)	0.316
HBs Ag-positive		14 (14.3)	8 (12.9)	6 (16.7)	0.607
Anti-hepatitis C virus positive		30 (30.6)	16 (25.8)	14 (38.9)	0.175
Alcohol abuse		29 (29.9)	18 (29.0)	11 (31.4)	0.804
Smoking		30 (30.9)	23 (37.1)	11 (31.4)	0.08
**Main reason for ICU admission**					
Acute respiratory failure		38 (38.8)	24 (38.7)	14 (38.9)	0.888
Sepsis		11 (11.2)	6 (9.7)	5 (13.9)	0.57
Severe neurologic disorders		25 (25.5)	15 (24.9)	10 (27.8)	0.542
Miscellaneous		24 (24.4)	17 (27.4)	7 (19.4)	-
**Type of diagnosis at ICU admission**					
	Opportunistic infection	25 (25.5)	16 (25.3)	9 (25.7)	
	Severe bacterial infection	36 (36.7)	22 (34.9)	14 (40)	
Related to HIV	HAART-induced complications	6 (6.1)	5 (7.9)	1 (2.8)	0.975
	Non infectious disease related to HIV	15 (15.3)	7 (11.1)	8 (22.8)	
	Total	82 (83)	50 (79.3)	32 (91.4)	
Unrelated to HIV		16 (16.3)	13 (20.7)	3 (8.6)	0.169
**Details of opportunistic infection at the admission**					
*Pneumocystis jirovecii* pneumonia		11 (11.2)	6 (9.7)	5 (13.9)	
Tuberculosis		1 (1)	0	1 (2.8)	
Cerebral toxoplasmosis		6 (6.1)	5 (8.1)	1 (2.8)	-
Cryptococcosis		3 (3.1)	2 (3.2)	1 (2.8)	
Others		4 (4)	3 (4.8)	2 (5.6)	
No opportunistic infection		73 (74.5)	46 (74.2)	27 (75)	0.621
**Life-supporting procedures in ICU**					
Mechanical ventilation during the 24 first hours		59 (60.2)	28 (45.2)	31 (86.1)	<0.001
Duration of mechanical ventilation, days (mean ± SD)		9.59 ± 17.0	7.61 ± 15.4	13 ± 19.4	0.003
Renal replacement therapy		15 (15.3)	7 (11.3)	8 (22.2)	0.147
Duration of renal replacement therapy, days (mean ± SD)		1.7 ± 6.4	1.3 ± 5.3	2.3 ± 8.1	0.37
Vasopressors		51 (52)	21 (33.9)	30 (83.3)	<0.001
Duration of vasopressors, days (mean ± SD)		3.8 ± 6.6	2.6 ± 4.5	5.9 ± 8.8	<0.001
Length of stay in ICU, days (mean ± SD)		14 ± 17.5	13.4 ± 16.1	15.0 ± 19.9	0.431
**Severity score (at 24 h)**					
SAPS II (mean ± SD)		53.8 ± 20.7	46.5 ± 13.5	66.2 ± 24.9	<0.001
**Biological data at admission**					
Albumin, g/L (mean ± SD)		31.1 ± 14.2	32.8 ± 14.8	27.6 ± 12.4	0.125
Lactic acid, mmol/l (mean ± SD)		2.9 ± 2.9	2.1 ± 1.9	4.4 ± 3.9	<.001
Thrombin time,% (mean ± SD)		68.4 ± 22.9	72.5 ± 22	61.6 ± 23	0.034

ICU, intensive care unit; SD, standard deviation; SAPS, Simplified Acute Physiology Score; HBs Ag, hepatitis B virus surface antigen; HAART, highly active antiretroviral therapy.

*p is significant.

HIV characteristics are presented in Tables 1 and 2. Eighty-two (83.6%) admissions were related to HIV infection: 25 admissions (25.5%) were due to an AIDS-associated illness (11 Pneumocystis pneumonia, 6 cerebral toxoplasmosis, 1 tuberculosis, 3 cryptococcosis, 2 non-tuberculosis mycobacterium infections, and 2 severe cytomegalovirus reactivations); 15 (15.3%) to non-infectious-complications of HIV infection (5 hemolytic and uremic syndromes, 2 HIV-cardiomyopathy decompensations, 2 lymphomas, 2 Castleman diseases, and 4 glomerulonephritis); 6 complications induced by specific treatments: 1 hepatitis related to antituberculous, 1 methemoglobinemia with disulone, 1 severe thrombopenia due to cotrimoxazole, and 3 complications related to HAART (1 Lyell disease due to nevirapine, 1 tubulopathy due to tenofovir, and 1 pancreatitis related to didanosine), and 36 patients (36.7%) were admitted to ICU for severe bacterial infections. Sixteen patients (16.3%) had an HIV-unrelated event (severe asthma, brain hemorrhage, medullar ischemia, ischemic heart failure, severe metabolic disorders). Overall, 44 of 98 patients (45%) received HAART at the time of ICU admission. HAART was interrupted for more than 72 h during ICU stay in 24 patients for nonspecified reasons. Nine patients received HAART for the first time in ICU. Eight patients (8%) had their HIV-infection discovered after ICU admission.

**Table 2:** Demographic and baseline clinical characteristics of HIV-infected patients admitted to the ICU, stratified according to immunovirological statuses

		**Patients with complete immunovirological status available**				
	**Total**	**n = 76**				
		**CD4**^**high**^	**CD4**^**low**^	**CD4**^**low**^	**CD4**^**high**^	***p *value**
	**n = 98**	**VL**^**high**^	**VL**^**high**^	**VL**^**low**^	**VL**^**low**^	
**Variable (n,%)**		n = 14	n = 40	n = 10	n = 12	
Males	69 (70.4)	8 (57.1)	28 (70)	8 (80)	10 (83.3)	0.496
Age, yr (mean ± SD)	43.1 (10.7)	42.2 (8.5)	40.1 (8.7)	46.8 (16.7)	54.7 (10.5)	<0.001
**Time since HIV diagnosis**						
Less than 6 months	17 (17.3)	1 (7.1)	8 (20)	1 (10)	0 (0)	
Intermediate	14 (14.3)	4 (28.6)	6 (15)	2 (20)	2 (16.7)	0.62
More than 5 years	67 (68.4)	9 (64.3)	26 (65)	7 (70)	10 (83.3)	
HIV diagnosis in ICU	8 (8.2)	0 (0)	6 (15)	0 (0)	0 (0)	0.217
**Comorbidities**						
HbsAg-positive.	14 (14.3)	3 (21.4)	6 (15)	0 (0)	1 (8.3)	0.532
Anti-hepatitis C virus positive	30 (30.6)	10 (71.4)	7 (17.5)	4 (40)	2 (16.7)	0.001
HbsAg + antihepatitis C virus positive	7 (7.1)	3 (21.4)	2 (5)	0 (0)	0 (0)	0.117
Alcohol abuse	29 (30)	8 (57.1)	13 (32.5)	2 (20)	2 (16.7)	0.137
Tobacco	30 (31)	8 (57.1)	15 (37.5)	1 (10)	3 (25)	0.099
Past medical history of cancer	13 (12.6)	0 (0)	3 (7.5)	4 (40)	2 (16.7)	0.016
**Main reasons for ICU admission**						
Acute respiratory failure	38 (38.8)	8 (57.1)	16 (40)	2 (20)	3 (25)	
Sepsis	11 (11.2)	1 (7.1)	3 (7.5)	1 (10)	2 (16.7)	0.749
Severe neurologic disorders	25 (25.5)	2 (14.3)	11 (27.5)	4 (40)	3 (25)	
Miscellaneous	24 (24.5)	3 (21.5)	10 (25)	3 (30)	6 (33.3)	
**Opportunistic infections**						
No opportunistic infection	73	14 (100)	25 (62.5)	10 (100)	11 (91.7)	0.002
*Pneumocystis jirovecii* pneumonia	11 (11.2)	0 (0)	5 (12.5)	0 (0)	1 (8.3)	
Tuberculosis	1 (1)	0 (0)	1 (1)	0 (0)	0 (0)	-
Toxoplasmosis	6 (6.1)	0 (0)	4 (10)	0 (0)	0 (0)	
Cryptococcosis	3 (3)	0 (0)	2 (5)	0 (0)	0 (0)	
Reactivation of cytomegalovirus (antigenemia positive)	15 (15.3)	0 (0)	10 (25)	0 (0)	1 (8.3)	0.054
**ICU data**						
SAPS II score (median)	49.5	52	48.5	56	49.5	0.522
APACHE II score (median)	17.5	16.5	18	20	14.5	0.509
Albumin, g/dL (median)	26.1	32.1	28.8	30.7	42.1	0.169
Mechanical ventilation in the first day	59 (60.2)	10 (71.4)	19 (47.5)	8 (80)	8 (66.7)	0.176

APACHE, Acute Physiology and Chronic Health Evaluation; SAPS, Simplified Acute Physiology Score; ART, antiretroviral therapy; HIV, human immunodeficiency virus; HCV, hepatitis C virus; HbsAg, hepatitis B virus surface antigen; ICU, intensive care unit; CD4, lymphocyte T CD4(+); CD4^high^, ≥200 cell/mm^3^; CD4^low^, <200 cell/mm^3^; VL, viral load; VL^high^, ≥10^3 ^copies/ml; VL^low^, <10^3^ copies/ml; HAART, highly active antiretroviral therapy.

*p is significant.

Complete immunovirological statuses were determined in 76 patients (77.6%): 4 patients had no plasma HIV RNA and CD4 count, 1 had plasma HIV RNA but no CD4 cell count, and 17 had a CD4 cell count but no plasma HIV RNA. Clinical characteristics of these patients are presented in Table 3. Patients were older in group [CD4^*low*^ + VL^*low*^] or [CD4^*high*^ + VL^*low*^] than in group [CD4^*high*^ + VL^*high*^] and in group [CD4^*low*^ + VL^*high*^]. Coinfection with hepatitis C virus was present in 71% of patients in group [CD4^*high*^ + VL^*high*^] versus <40% in the other groups (*p* = 0.001). In group [CD4^*low*^ + VL^*low*^], all patients were treated with HAART for a long time and viral load was <3 log_10_/ml for at least 1 year before ICU admission. None of these patients had opportunistic infection at admission in ICU, and most of them were hospitalized for various reasons, such as suicide, gastrointestinal hemorrhage associated with end-stage liver cirrhosis, septic shock after chemotherapy for non-classifying-AIDS cancers, malignant hypercalcemia, or acute lung injury. In this group, five patients left ICU, but the only patient discharged alive from the hospital died 6 months later.

**Table 3:** Specific characteristics of the 98 HIV-infected patients

**Variables (%)**		**Total**	**Survived the ICU**	**Died in the ICU**	***p *value**
		**(n = 98)**	**(n =62)**	**(n =36)**	
**HAART**					
No past history of HAART		24 (24.5)	16 (25.8)	8 (22.2)	0.669
No HAART at the admission + no introduction in ICU		45 (45.9)	28 (45.2)	17 (47.2)	
No HAART at the admission + introduction in ICU		9 (9.2)	7 (11.3)	2 (5.6)	0.106
HAART active at the admission and pursued		20 (20.4)	16 (25.8)	4 (11.1)	
HAART active at the admission and stopped		24 (24.5)	11 (17.7)	13 (36.1)	
Introducing or pursuing HAART in ICU		29 (29.6)	23 (37.1)	6 (16.7)	0.032
**Immunovirological data**					
Mean ± SD CD4 (/mm^3^) §		173.5 ± 192	176.3 ± 197.1	168.7 ± 185.8	0.701^¶^
Mean ± SD HIV RNA VL (10^3^ copies/ml)¶		274. 8 ± 664.9	282.6 ± 709.5	261.1 ± 591	0.975
CD4^high^ (≥200/mm^3^)		30 (32.3)	20 (33.9)	10 (29.4)	0.656
CD4^low^ (<200/mm^3^)		63 (67.7)	39 (66.1)	24 (70.6)	
Combined	CD4^high^ + VL^high^	14 (18.4)	9 (18.4)	5 (18.5)	
subgroups	CD4^high^ + VL ^low^	12 (15.8)	10 (20.4)	2 (7.4)	0.429
	CD4^low^ + VL^high^	40 (52.6)	25 (51)	15 (55.6)	
	CD4^low^ + VL^low^	10 (13.2)	5 (10.2)	5 (18.6)	

^§^93 patients; ^¶^77 patients.

ICU, intensive care unit; CD4, lymphocyte T CD4(+); CD4^high^, ≥200 cell/mm3; CD4^low^, <200 cell/mm3; VL, viral load; VL^high^, ≥1,000 copy/ml; VL^low^, <1,000 copy/ml; HAART, highly active antiretroviral therapy.

*p is significant.

#### In-ICU mortality and 1-year mortality

Thirty-six patients (36.7%) died in the ICU and 12 patients (12.2%) died in the hospital after ICU discharge (Figure 1). From 1997 to 2008, ICU mortality decreased, whereas severity score remained constant and patients were older, but those trends were not significant (Table 4). In univariate analysis, factors significantly associated with ICU mortality were the use of vasopressive agents or mechanical ventilation, the arterial lactic acid value, and the severity scores (SAPS II or APACHE II) at admission. In our study, neither CD4 T-cell count (even for patient with very low cell count as <50/mm^3^) nor anteriority of HIV diagnosis, viral load, HAART at admission, reason for admission, and immunovirological status were significantly associated with ICU mortality. Introducing or continuing HAART in ICU was significantly associated with a better outcome (Table 3).

**Table 4:** Evolution of ICU mortality, age, and SAPS II score of HIV-infected patients admitted to the ICU

**Period**	**All period**	**1997–2000**	**2001–2004**	**2005–2008**	***p *value**
	**n = 98**	**n = 29**	**n = 37**	**n = 32**	
Died in ICU (n,%)	36 (36.7)	13 (44.8)	13 (35.1)	10 (31.2)	0.529
Died before hospital discharge (n,%)	47 (52.8)	13 (44.8)	20 (54.0)	14 (43.7)	0.832
Died at one year* (%)	55	50.4	59.6	52.7	0.843
Age (means, SD)	42.8 ± 10.6	36.1 ± 6.1	44.6 ± 10.1	47.6 ± 11.6	<0.001
SAPS II (means, SD)	53.2 ± 20.3	52.9 ± 25.7	51.7 ± 15.0	56.9 ± 21.5	0.406

ICU, intensive care unit; SD, standard deviation; SAPS, Simplified Acute Physiology Score.

*Percentage extrapolated from survival curves of Kaplan-Meier and compared using a log-rank test.

After multivariate logistic regression analysis including the SAPS II score, the only independent predictors of ICU mortality were the use of vasopressive agents (OR, 3.78; 95% CI, 1.11-12.86, *p* = 0.03) and the SAPS II score (OR, 1.04; 95% CI, 1.00-1.08; *p* = 0.03), whereas introducing or continuing HAART in ICU was protective (OR, 0.28; 95% CI, 0.08-0.94; *p* = 0.04; Table 5).

**Table 5:** Univariate and multivariate analysis of variables associated with in-ICU and 1-year mortality

**Variables**		**ICU mortality**		**One-year mortality**	
		**Crude odds ratio**	**Adjusted odds ratio**	**Crude hazard ratio**	**Adjusted hazard ratio**
		**OR [95% CI] *p***	**OR [95% CI] *p***	**HR [95% CI] *p***	**HR [95% CI] *p***
**HAART**					
No HAART at admission + no introduction in ICU		1.00 [−] -	-	1.00 [−] -	1.00 [−]
No HAART at admission + introduction in ICU		0.471 [0.087-2.533] 0.380	-	0.807 [0.277-2.355] 0.695	0.166 [0.043-0.642] 0.009
HAART active at admission and pursued		0.412 [0.118-1.438] 0.164	-	0.948 [0.446-2.014] 0.889	1.519 [0.519-4.444] 0.446
HAART active at the admission and stopped		1.947 [0.713-5.312] 0.193	-	1.827 [0.963-3.466] 0.065	1.758 [0.633-4.887] 0.279
Introducing or pursuing HAART in ICU		0.339 [0.123-0.937] 0.037	0.278 [0.082-0.939] 0.039	0.72 [0.39-1.33] 0.294	-
**Immunovirological status**					
CD4 T-cell count <200 /mm^3^		1.332 [0.542-3.273] 0.532	-	1.348 [0.73-2.489] 0.34	-
HIV RNA load >1,000 copy/mL		1.153 [0.503-2.645]- 0.737	-	0.935 [0.541-1.616] 0.809	-
	CD4^high^ + VL ^low^	1.00 [−] -	-	1.00 [−] -	1.00 [−] -
Combination	CD4^high^ + VL^high^	2.778 [0.428-18.037] 0.857	-	2.736 [0.707-10.594] 0.145	2.344 [0.442-12.434] 0.317
	CD4^low^ + VL^high^	3.000 [0.578-15.583] 0.659	-	2.583 [0.77-8.670] 0.124	5.19 [1.328-20.279] 0.018
	CD4^low^ + VL ^low^	5.000 [0.704-35.493] 0.208	-	7.411 [2.02-27.195] 0.002	4.714 [1.178-18.867] 0.028
**Comorbidity**					
Hepatitis C virus infection		1.83 [0.760-4.406] 0.178	-	1.469 [0.829-2.603] 0.1879	3.268 [1.29-8.278] 0.012
**In-ICU data**					
Use of vasoactive drugs		9.762 [3.512-27.132] <0.001	3.779 [1.11-12.861] 0.033	4.2 [2.257-7.815] <0.001	3.68 [1.394-9.716] 0.008
Mechanical ventilation		7.529 [2.585-21.923] <0.001	3.317 [0.955-11.521] 0.059	3.139 [1.64-6.007] <0.001	-
Albumin		0.97 [0.926-1.015] 0.19	-	0.968 [0.937-1.001] 0.058	-
Acid lactic in the first 24 h		1.326 [1.104-1.593] 0.002	-	1.205 [1.109-1.31] <0.001	-
SAPS II		1.06 [1.029-1.091] <0.001	1.04 [1.003-1.077] 0.032	1.065 [1.048-1.084] <0.001	1.09 [1.057-1.124] <0.001

ICU, intensive care unit; CD4, lymphocyte T CD4(+); CD4^high^, ≥200 cell/mm^3^; CD4^low^, <200 cell/mm^3^; VL, viral load; VL^high^, ≥10^3^ copy/ml; VL^low^, <10^3^ copy/ml; HAART, highly active antiretroviral therapy; OR, odds ratio; HR, hazard ratio; CI, confidence interval; SAPS, Simplified Acute Physiology Score; HBs Ag, hepatitis B virus surface antigen.

*p is significant.

**Figure 2:**
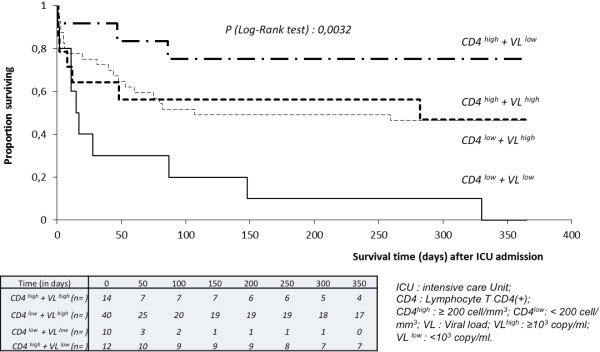
Kaplan-Meier survival curves illustrating time to death during the first year after admission in ICU according to the immune-virological status.

Eight of the 50 patients who were discharged from hospital were lost to follow-up. At 1 year after ICU admission, 34 patients (34.7%) were alive (Figure 1). With univariate analysis, 1-year mortality was significantly higher for patients classified in the group [CD4^*low*^ + VL^*low*^] compared with all other groups (Table 5; Figure 2). Introducing or continuing HAART in ICU was not significantly associated with a better outcome.

Using Cox proportional hazard models, including the SAPS II, the factors independently associated with 1-year mortality were: belonging to the group [CD4^*low*^ + VL^*high*^] vs. [CD4^*high*^ + VL^*low*^] (HR, 5.19; 95% CI, 1.33-20.28; *p* = 0.02) and to the group [CD4^*low*^ + VL^*low*^] vs. [CD4 ^*high*^ + VL^*low*^] (HR, 4.71; 95% CI, 1.18-18.87; *p* = 0.03), coinfection with hepatitis C virus (HR, 3.27; 95% CI, 1.29-8.28; *p* = 0.01), the use of vasopressive agents (HR, 3.68; 95% CI, 1.39-9.72; *p* ≤0.01), and the SAPS II score (HR, 1.09; 95% CI, 1.057-1.124; *p* ≤0.0001; Table 5). Introducing HAART in a patient with no HAART at admission was associated with a better outcome (HR, 0.17; 95% CI, 0.04-0.64; *p* ≤0.01).

## Discussion

In this study, we observed a high rate of mortality in ICU, a majority of admissions not primarily related to opportunistic infections and only a few discoveries of HIV infection after ICU admission. In this HIV-infected population, we showed that mortality in ICU and during the first year following admission were related to the acute illness severity and immunovirological statuses at ICU admission and that introduction or pursuit of HAART seemed to be associated with a better outcome. The 1-year mortality contrasted with the ICU mortality (62.2% and 36.7% respectively), suggesting that the long-term survival of critically ill HIV-infected patients was still poor. Our in-ICU mortality was similar to other centers in industrialized countries [12,16,27,28], but our 1-year survival rate was lower compared with the few studies reporting long-term outcomes, in which antiretroviral-naïve subjects were the vast majority [15,17]. This last point can be explained by the fact that the HIV-infected subjects in our study had a long history of HIV infection and treatment and that morbid outcomes after ICU discharge were independent of immune restoration under HAART (i.e., non-AIDs defining cancers, decompensated cirrhosis).

As in several other studies, use of vasopressive agents, mechanical ventilation, and severity scores at the admission were the main prognostic factors of ICU mortality [9,10,18,19,28].

HIV-infected patients admitted to ICU do not form a homogenous population and should be considered according to their immunovirological status. For example, patients with HAART failure and deep immunological deficiency, patients with AIDS classifying diseases but who never had antiretroviral therapy, or patients in the early stage of the HIV infection with high CD4 cell count will probably have very different outcomes. These different situations are not entirely captured by analyzing separately plasma HIV RNA viral load and CD4+ cell count. In our study, death at 1 year after admission was not directly related to a CD4+ cell count <200/mm^3^. We thus decided to classify patients into four groups according to viral load and CD4 counts at the admission and showed that patients from the groups [CD4^*low*^ + VL^*low*^] or [CD4^*low*^ + VL^*high*^] had a very bad outcome at 1 year compared with the group [CD4^*high*^ + VL^*low*^]. The group [CD4^*low*^ + VL^*low*^] contained patients with viral replication suppressed by HAART but without immune reconstitution and the group [CD4^*low*^ + VL^*high*^] patients with HAART failure, whereas the group [CD4^*high*^ + VL^*low*^] corresponded to patients with a good response to HAART. To our knowledge, combined plasma HIV RNA viral load and CD4+ cell count have never been evaluated as prognostic factor in HIV-infected patients admitted to ICU.

In the general HIV-infected population presenting an opportunistic infection, early HAART resulted in less AIDS progression/death with no increase in adverse events or loss of virologic response compared to deferred HAART [29]. Moreover, it is now recommended to prescribe HAART in the majority of HIV-infected patients even for high CD4 level [26]. Management of antiretroviral therapy is a challenge in ICU, and introducing HAART in a patient with no HAART at admission is a difficult decision. There are no pharmacologic and pharmacokinetic data concerning antiretroviral therapy in hemodynamically unstable patients. Drug-to-drug interactions are expected with antiarrhythmics, anticonvulsants, antifungal, antibiotics, gastrointestinal drugs, or sedative drugs, such as midazolam. Only AZT can be used for intravenous administration, and pharmacokinetics of administration using nasogastric tubes for others compounds are uncertain and could lead to poor drug absorption and further development of resistance. In our study, we showed that introducing or continuing HAART in HIV-infected patients in ICU seems to be protective in the short-term and introducing HAART in a patient with no HAART at admission seems to be associated with a better long-term outcome. One must probably discriminate HIV-related situations in which immune restoration is crucial for clinical response or to prevent other complications (especially in case of long period of ICU) from other situations.

Only four retrospective studies analyzed the impact of HAART in ICU and showed discordant results. A study of 281 patients admitted to ICU in the San Francisco General Hospital found that HAART use at ICU admission was not associated with a better hospital survival but was associated with predictors of better outcome, such as higher serum albumin concentrations [8]. The second study from São Paulo University Hospital analyzed predictive factors of mortality for 278 HIV patients admitted to ICU; 193 of them (69.4%) died before 6 months [17]. They demonstrated that HAART use in ICU was negatively predictive of 6-month mortality, especially if this therapy was introduced during the first 4 days of admission in ICU. However, contrasting with our and other studies, 80.6% of the ICU admissions were due to AIDS-defining conditions, and all subjects had very low CD4 counts. The third study from Saint Louis hospital in Paris [9] analyzed predictive factors of mortality for 284 HIV patients admitted to ICU and showed that comorbidities and organ dysfunctions, but not HIV-related variables, were associated with death. Among the 233 (82%) patients with known HIV infection before ICU admission, 64% were on HAART but HAART use at ICU admission was not associated with ICU mortality.

The most recent study, from the University Medical Center Utrecht in Netherlands [21], analyzed characteristics from patients admitted to the ICU during the pre-HAART era (n = 47), and during the HAART period (1996–2008, n = 80). The 1-year mortality in the HAART era decreased to 51%. Predictors of short- and long-term (1 year and 5 years) mortality in the HAART era were older age, APACHE II score > 20, and the use of mechanical ventilation, but neither HIV-related factors nor the use of HAART on ICU admission were significant.

Our study has several limitations. First, the design was retrospective. All patients were managed in two different ICU without standardized, written protocols, but all patients had a follow-up in the same Infectious Diseases Department and no data of ICU concern were missing. Loss of follow-up is less than 10%, which is usual in this pathology. During this period, we recorded only 104 admissions in this two medical ICU, but we have no data about HIV-infected patients hospitalized in surgical ICU. Second, the study period is very long (11 years), comprising progress in critical care practices during the past decade: use of low tidal volumes for patients with respiratory failure, adjuvant corticosteroids for certain conditions, and early goal-directed therapy for sepsis. Even if the admission policy of HIV-infected patients remains constant in our centers, antiretroviral therapy also has evolved and new combinations are supposed to enhance efficiency with fewer adverse events. However, number of admissions, age, and severity scores throughout the different periods were not significantly different (Table 4). Third, numbers of patients in each immunovirological subgroup were too small to discriminate patients who really benefited from HAART use in ICU. Our results suggest that introduction of HAART is beneficial in some patients in the ICU, but this protective effect may be simply an association rather than a causative factor.

## Conclusions

In our population of HIV-infected patients admitted to ICU, the ICU and 1-year mortality are related to acute illness severity and immunovirological status at admission. Complementary studies are necessary to identify HIV-infected patients who benefit from HAART use in ICU according to immunovirological status and the reasons of ICU admission.

## Competing interests

The authors declare that they have no competing interests.

## Authors’ contributions

DM, VLM, and PC designed the study project. DM collected the data and wrote the article. TM made the statistical analysis. All authors have reviewed and approved the manuscript. DM and PC are the guarantors of the paper, taking responsibility for the integrity of the work as a whole, from the inception of the project to the published article. All authors read and approved the final manuscript.
